# Intelligent management and legal regulation of enterprise green supply chain by fuzzy comprehensive evaluation

**DOI:** 10.1016/j.heliyon.2024.e39929

**Published:** 2024-10-29

**Authors:** Junfeng Wang

**Affiliations:** School of Law, Liaoning Normal University, Dalian, 116083, China

**Keywords:** Fuzzy comprehensive evaluation, Green supply chain, Supply chain intelligent management risk, Legal supervision legal regulation

## Abstract

This study aims to evaluate the risk levels of intelligent management in Green Supply Chain Management (GSCM) through the application of the Fuzzy Comprehensive Evaluation (FCE) model. It begins by outlining the structure of the green supply chain (GSC), encompassing various components such as suppliers, manufacturers, and distributors, while incorporating considerations of environmental and sustainability factors. Data is collected from Company Y through a questionnaire survey, and the FCE model is employed to assess the risk levels of intelligent management within GSCM. The study identifies that Company Y faces high-risk factors in supply chain management, including customer demand fluctuations, inadequate supplier capacity, and reliance on exclusive suppliers, with a risk rating of 4. Additionally, moderate-risk factors, such as partnerships, policies, cash flow, technological capabilities, and internal production management, were rated at a risk level of 3. Based on the risk assessment results, the study proposes several recommendations for improvement. These include reinforcing the enforcement of environmental policies, establishing mechanisms for supply chain information transparency, and promoting clean production technologies. These recommendations are intended to assist the company in better adhering to environmental regulations and fostering sustainable supply chain development. The study's innovation lies in the successful integration of the FCE model into the supply chain risk assessment framework, effectively combining subjective evaluations with objective data to improve the accuracy of the assessment. The findings provide scientific evidence for companies to manage risks and formulate strategies, while also offering new insights into the multidimensional nature of supply chain risks, encompassing social, economic, environmental, and technological dimensions.

## 1 Introduction

As technology and human civilization continue to advance, the global economy is experiencing rapid transformation, accompanied by increasingly prominent ecological and environmental challenges. The acceleration of globalization has posed significant threats to natural resources and the ecological environment. In response to the constraints of resource scarcity and environmental degradation, and to shift from a model characterized by high input, consumption, and emissions towards a healthier and more sustainable economic trajectory, the pursuit of a “green revolution” has emerged as a focal point since the 1990s. This movement has garnered widespread attention and support from governments and enterprises worldwide [1,2]. Green environmental protection has become a central issue in contemporary discussions and has gradually entered the public consciousness. Driven by global environmental challenges and economic integration, Green Supply Chain Management (GSCM) has emerged as a viable strategy to address enterprise-related pollution concerns. However, the supply chain also introduces additional risks and competitive pressures for enterprises. These risks propagate throughout the supply chain, creating vulnerabilities and hindering the efficiency of Supply Chain Management (SCM), which can result in various forms of losses for supply chain entities [3]. In this context, the present study focuses on the fuzzy evaluation of GSCM risks.

In Supply Chain Management (SCM), the risk and performance evaluation of a company's Green Supply Chain (GSC) involves numerous subjective and objective indicators, which are often characterized by ambiguity and uncertainty, making precise numerical expression ineffective. Fuzzy evaluation theory provides a method for handling fuzzy and uncertain information, allowing for a more comprehensive and accurate risk assessment by integrating both qualitative and quantitative factors. Additionally, GSCM must adhere to various environmental regulations and social responsibility standards to ensure compliance and avoid environmental pollution and social responsibility issues within the supply chain. These regulations vary by region, requiring companies to adjust their supply chain strategies in accordance with local laws. Therefore, understanding and adapting to these regulations is a crucial context for GSCM research.

In existing supply chain management research, the application of fuzzy logic has been widely discussed, but it has mainly focused on identifying and evaluating individual risk factors. The Fuzzy Comprehensive Evaluation (FCE) model proposed in this study introduces significant innovations and improvements in the following areas. Firstly, comprehensive evaluation of multidimensional risk factors: this model not only considers individual risk factors within the supply chain but also evaluates multiple risk factors such as supplier capability, policy changes, and market demand fluctuations. This multidimensional approach offers a more holistic reflection of the risks present in supply chain management. Secondly, effective integration of subjective and objective factors: traditional fuzzy evaluation methods often struggle to balance subjective judgment and objective data. The proposed FCE model effectively integrates subjective evaluations and objective data by combining expert experience with historical data, improving the accuracy and reliability of the risk assessment. Thirdly, dynamic risk assessment mechanism: this model is not only suitable for static risk assessments but also dynamically reflects changes in risks within supply chain management. By monitoring and analyzing key indicators in real-time, it allows for timely adjustments in risk assessments, providing more immediate references for enterprise decision-making. Lastly, integration of legal regulations and risk management: in existing research, legal regulations and risk management are often discussed separately. The proposed FCE model incorporates regulatory factors into the risk assessment framework, helping companies optimize supply chain management while complying with environmental regulations and achieving sustainable development.

To integrate the application of fuzzy evaluation in corporate GSC intelligent management and legal regulations, this study is structured as follows. Section 1 introduces the background, objectives, and overall structure of the study. Section 2 provides a review of previous research contributions and achievements, outlining the current state of the field. Section 3 discusses fuzzy evaluation methods, the risks associated with intelligent supply chain management, and the relevant legal regulations affecting GSC intelligent management. The study validates the application of fuzzy evaluation through a risk assessment framework specifically designed for GSC intelligent management. Section 4 presents and validates the results obtained from combining fuzzy evaluation with questionnaire data, offering recommendations and strategies to enhance corporate GSCM while ensuring compliance with applicable legal regulations. It also initiates a discussion on these topics. Section 5 concludes by summarizing the study's contributions and proposing directions for future research. This study introduces the fuzzy evaluation method and integrates it with an intelligent management model to assess the risk levels of corporate GSC. Furthermore, this innovative approach addresses uncertainty and ambiguity in the risk assessment process, improving accuracy and reliability. By promoting innovation, sustainable development, and legal compliance in GSC, this study provides valuable insights and guidance for enterprises in the field of GSCM.

## 2 Literature review

Considerable scholarly attention has been dedicated to exploring various dimensions of corporate GSCM. Dong et al. examined the impact of GSCM on clean technology innovation in Chinese enterprises, comparing its forward and backward effects across different industries. Their findings indicated that GSCM significantly benefited enterprise clean technology innovation, and this relationship remained robust across a series of sensitivity tests [4]. Tarigan et al. investigated the effect of enhanced enterprise resource planning (ERP) on performance through GSCM, supplier integration, and internal integration. They demonstrated that GSCM, as well as internal and supplier integration, mediated the impact of enhanced ERP on firm performance. The relationships between these four constructs were analyzed, with each serving as a mediating variable [5]. Jo and Kwon explored the structural relationship between environmental cooperation, green innovation capability, and performance to identify the factors influencing the GSCM performance of Korean manufacturing Small and Medium-sized Enterprises (SMEs). They concluded that environmental cooperation within the GSC framework was a key driver of green innovation capability in Korean manufacturing SMEs [6]. Le, using data from 218 building material manufacturers in Vietnam, employed the structural equation model (SEM) to investigate the influence of GSCM on enterprise performance. His study revealed that green design and manufacturing had a significant positive impact on three performance outcomes [7]. Bu et al. explored the relationship between environmental orientation and enterprise performance, focusing on the mediating role of GSCM. Both internal and external environmental orientation were positively correlated with the three elements of GSCM (environmental selection, monitoring, and cooperation with suppliers), which, in turn, were positively correlated with enterprise performance [8]. Finally, Liu et al. focused on optimizing the treatment and transportation paths for industrial hazardous waste by environmental protection enterprises, with a particular emphasis on the green procurement process. Their study linked the procurement management of environmental enterprises with production under GSCM, achieving effective storage and disposal of hazardous waste [9].

In terms of the theoretical background of GSCM, Umar et al. (2022) underscored the importance of environmental sustainability in SCM and proposed a conceptual model for GSC, which laid the groundwork for subsequent research [10]. This model encompassed the entire supply chain process—from raw material acquisition to product recovery—highlighting the integration of environmentally sustainable practices. Khan et al. (2023) further examined the impact of green practices on business performance, emphasizing the crucial role of collaboration between environmental stakeholders and supply chain partners in improving environmental performance [11]. Regarding the theoretical foundation of fuzzy evaluation methods, Riaz et al. (2021) introduced fuzzy set theory, which provides robust mathematical tools for addressing uncertainty and vagueness. Building on this theory, fuzzy evaluation methods transform qualitative descriptions into fuzzy numerical values and use fuzzy logic for reasoning and assessment, thereby effectively managing uncertainty in multi-criteria decision analysis [12]. Aydemir et al. (2020) developed the FCE method and demonstrated its application in multi-criteria decision support systems through practical case studies [13]. Furthermore, Narassima et al. (2024) emphasized the potential of fuzzy logic in addressing supply chain uncertainties, providing a theoretical basis for subsequent research on supply chain challenges using fuzzy evaluation methods [14]. Tronnebati et al. (2022) applied fuzzy evaluation methods to the selection of supply chain partners, showcasing the effectiveness of these methods in practical SCM decision-making processes [15]. In summary, the theoretical foundation of this study is strongly supported by extensive academic research. The integration of GSCM theory and practice highlights the vital role of environmental sustainability in modern SCM, while fuzzy evaluation methods serve as effective tools for addressing uncertainty and ambiguity within the supply chain. By synthesizing these theories and methods, this study seeks to offer new insights and solutions for risk assessment in GSCM.

A comparative analysis of various methods for assessing risks in GSC intelligent management is presented in Table 1.

**Table 1:** Comparison of risk assessment methods for the GSC intelligent management.

Method	principle	Advantage	Limitation
Multi-attribute Decision [16]	Risk indicators weighted combination	Careful consideration of multiple factors	Weighting may excessively impact results
Matrix Method [17]	Risk-categorized and cross-matched	Simple, user-friendly, intuitive	Inability to handle fuzzy and uncertain information
Statistical Analysis [18]	Risk prediction through historical data	Data analysis, risk quantification, statistical support	Reliance on historical data, inability to cope with future uncertainty
AI & ML [19]	Learning risk patterns from data, prediction, and classification	Detection of hidden patterns and trends, suitable for large-scale data analysis	Requires substantial historical data for training, complex model resulting in difficult interpretation of results

In Table 1, the current methods for managing GSCM include multi-attribute decision-making, risk matrices, statistical analysis, and AI & ML techniques.

As a critical strategy integrating environmental sustainability with SCM, GSCM has garnered significant attention. The work of various scholars provides essential theoretical frameworks and empirical evidence, laying a solid foundation for this study. Younis et al. (2016) conducted an empirical study of China's manufacturing industry to examine the impact of GSCM implementation on enterprise performance. They found that practices such as green procurement and green production significantly improved environmental performance and competitiveness [20]. Abdallah et al. (2020) employed SEM to reveal the relationships between GSCM driving factors and enterprise performance, emphasizing the roles of policy regulations, market pressure, and supply chain collaboration [21]. Similarly, Maaz et al. (2022), through case analysis, demonstrated the application of GSC practices in the food industry, highlighting the importance of collaboration between supply chain partners and information sharing in the successful implementation of GSC [22]. Regarding fuzzy evaluation methods, Ayyildiz et al. (2023) proposed the Fuzzy Analytic Hierarchy Process (FAHP), providing a systematic approach to complex decision-making problems. This method combined the strengths of fuzzy set theory and the Analytic Hierarchy Process (AHP), effectively managing uncertainty and ambiguity, thus offering a robust theoretical tool for GSCM performance evaluation [23]. Ganguly et al. (2019) further advanced the FCE method, applying it to supply chain performance evaluation and demonstrating its effectiveness in processing fuzzy data [24]. Additionally, Hossain et al. (2023) used the fuzzy DEMATEL method to analyze the driving factors of GSCM and their interrelationships, identifying internal management, external environmental pressures, and supply chain partnerships as key drivers [25]. Building upon these contributions, this study focuses on applying fuzzy evaluation methods to GSCM performance evaluation, aiming to address existing research gaps in model development and empirical analysis. For example, Amin et al. (2022) studied GSC risk management and emphasized the role of fuzzy logic in uncertain environments, offering new perspectives and methodologies [26]. Furthermore, Carvalho et al. (2020) systematically reviewed recent advancements in GSCM, identifying future research directions and challenges, thus providing valuable insights for this study [27].

In the study by Mostafa et al. (2024), a hepatocellular carcinoma prediction method based on machine learning algorithms was proposed. By comparing the performance of different algorithms before and after feature dimensionality reduction, the effectiveness of feature reduction in improving the predictive model's performance was demonstrated [28]. Hassan et al. (2024) explored the application of language models and deep learning technologies in disease prediction, optimizing various hyperparameter tuning methods to predict diseases based on symptom descriptions, thereby supporting early detection and timely treatment [29]. Mamdouh Farghaly et al. (2023) and Mamdouh Farghaly et al. (2022) focused on the issue of feature selection, proposing a text classification feature selection technique based on association analysis that effectively reduces redundant information and improves classification accuracy [30,31]. Khairy et al. (2024) studied the effectiveness of oversampling and undersampling techniques in addressing class imbalance in cyberbullying detection, offering new approaches to improve the performance of classification algorithms on minority class samples [32]. Omar et al. (2024) combined 1D convolutional neural networks with long short-term memory networks to propose a novel optimization method for epilepsy seizure detection, significantly enhancing detection accuracy [33]. Farghaly et al. (2020a) combined association rule mining with support vector machines to propose an efficient associative classification method, improving classification accuracy and efficiency by selecting the optimal feature subset and reducing the number of generated rules [34]. Farghaly et al. (2020b) introduced a filter-based approach, automatically determining the threshold for important feature subsets by combining concepts such as Chi-square, Relief-F, and mutual information, thus improving the predictive accuracy and execution efficiency of the model [35]. Badawy et al. (2021) focused on sustainable development in the educational sector, proposing an algorithm that extracts topics from text-based learning resources, links relevant resources, and generates interactive dynamic knowledge graphs, providing a new tool to facilitate self-learning and support sustainable development for communities and humanity [36]. These studies not only offer new theoretical perspectives and methods for supply chain management but also provide valuable references for practical risk assessment and management.

Although existing research has provided a solid theoretical and practical foundation for GSCM and fuzzy evaluation methods, certain gaps and limitations remain. Firstly, most studies focus primarily on enterprises within a single country or region, lacking broader applicability across different countries or industries. Furthermore, current research rarely addresses the risk assessment of intelligent management within GSCM, resulting in an insufficiently comprehensive analysis of supply chain uncertainties. Building upon existing research, this study employs a fuzzy evaluation model to assess the intelligent management risks in corporate GSC, distinguishing itself by effectively integrating the fuzzy evaluation model into the GSC risk assessment framework. This integration combines subjective and objective elements, enhancing the accuracy of the assessment results. For instance, the risk assessment identifies that Company Y's high-risk factors include fluctuations in customer demand, insufficient supplier capacity, and dependence on single suppliers. Medium-risk factors include partner relationships, policies, corporate cash flow, inadequate technical capabilities, and internal production management. Based on these risk assessment results, this study offers several recommendations, including the strict enforcement of environmental policies, the establishment of transparent GSC information mechanisms, and the promotion of clean production technologies. This study begins with the basic concepts and theories of GSCM, gradually introduces relevant research on fuzzy evaluation methods, and ultimately combines the two to explore their practical application and significance. Through a detailed analysis and synthesis of key literature, the study provides a solid theoretical foundation for future research, thereby enhancing its academic value and quality. This study not only expands the theoretical framework of GSCM and fuzzy evaluation methods but also empirically validates their effectiveness in practical applications. By conducting a systematic review of relevant literature, this study clarifies its position within the body of knowledge, emphasizing its contribution to ongoing discussions in the field. The results offer new perspectives for theoretical inquiry and provide a scientific basis for enterprise decision-making in GSCM practice. By applying fuzzy evaluation methods, this study thoroughly assesses the risks associated with intelligent management in corporate GSC, providing new insights and solutions for GSCM. Compared to previous models, the approach in this study is more effective and innovative in addressing supply chain uncertainties and ambiguities, offering practical guidance for enterprises in environmental regulation compliance and sustainable development.

The main differences and contributions of this study compared to existing literature in the field of GSCM are evident in several key areas. Firstly, this study utilizes the FCE model, which is not commonly employed in the existing literature. The FCE model assesses both individual risk factors within the supply chain and multiple risk factors, such as supplier capability, policy changes, and market demand fluctuations. This approach provides a more holistic risk assessment method by integrating subjective evaluations with objective data, thereby enhancing the accuracy and reliability of risk assessments. Secondly, this study introduces legal regulatory factors into the risk assessment framework, representing a significant advancement in the literature. By examining relevant environmental regulations and policies in China, the study offers specific legal regulatory recommendations, such as the strict enforcement of environmental policies, the establishment of GSC information disclosure mechanisms, and the promotion of clean production technologies. These recommendations aid enterprises in optimizing their SCM practices while ensuring compliance with environmental regulations. Thirdly, this study provides an empirical analysis to explore the specific manifestations and impacts of intelligent management risks within GSC. By analyzing the risk factors in Company Y's supply chain, the study identifies potential causes of high-risk factors and their specific impacts on enterprise operations. It also offers management recommendations, including enhancing supplier capabilities, diversifying supplier selection, and establishing long-term partnerships. Additionally, the study performs a cross-industry comparative analysis of GSCM across different industries and scales, offering targeted management strategies and recommendations. This cross-industry and cross-scale analysis provides new perspectives on the applicability and effectiveness of GSCM in various contexts. Finally, the study introduces methodological innovations by combining questionnaire surveys with fuzzy evaluation methods. This approach validates the effectiveness of the FCE model and enhances its reliability through expert consultations and email communications. The integration of qualitative and quantitative analyses presents a new research pathway for future studies. In summary, this study provides novel insights and tools across theoretical frameworks, methodologies, empirical analyses, and legal regulatory recommendations. It enriches academic discussions in the GSCM field and offers valuable guidance for enterprise practice.

## 3 Research methodology

### 3.1 Overview of GSCM

**Fig. 1:**
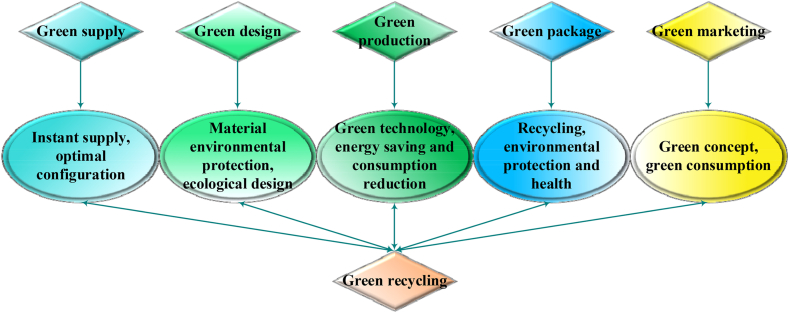
GSCM architecture.

In modern supply chain management, companies must possess the capability to quickly identify and respond to potential risks. For instance, by implementing advanced supply chain monitoring systems, businesses can track various links in the supply chain in real-time and promptly take corrective actions when issues arise. This rapid response mechanism not only helps mitigate losses caused by supply chain disruptions but also enhances overall operational efficiency. The GSC incorporates environmental considerations into traditional SCM [37,38]. Unlike traditional supply chains, which focus primarily on economic growth and development, GSC emphasizes a balance between economic advancement and environmental protection, aiming for a coordinated development model [39,40]. To achieve rapid and sustainable growth in a GSC, collaboration across all links in the supply chain is essential. Every participant in the supply chain must work towards sustainable development [41]. In line with the definition of each link in a traditional supply chain, the process of GSCM includes green supply, design, production, packaging, marketing, and the recycling of end-of-life products, as illustrated in Fig. 1 [42,43].

### 3.2 Construction of the FCE model

To ensure the effective application of the FCE model for assessing intelligent management risks in GSCM, this study commenced with an extensive review of relevant literature. Dong et al. (2021) highlighted that GSCM significantly impacts enterprises' clean technology innovation, particularly during the stages of supplier selection and product design [44]. This suggests that supplier capability and the sustainability of product design are crucial risk factors in GSCM. Tarigan et al. (2021) emphasized the influence of ERP on GSCM performance, notably through internal and supplier integration [45]. This underscores the necessity of considering the stability of internal processes and supplier relationships when constructing the FCE model. Jo and Kwon (2021) analyzed the structural relationship between environmental cooperation and green innovation capability, revealing that environmental cooperation is a critical driver of green innovation in GSCM [46]. Consequently, partner selection and cooperation models are also essential risk factors in the model. Tran et al. (2023) utilized data from Vietnamese building material manufacturers and found that green design and manufacturing positively impact GSCM performance [47]. This implies that green technologies and practices in the production process should be incorporated into the model as part of the risk assessment. Wiredu et al. (2024) explored the relationship between environmental orientation and firm performance, as well as the mediating role of GSCM [48]. This illustrates that environmental policies and practices should also be integrated into the risk assessment model. Based on the findings from these studies, this study identifies key risk factors for intelligent management in GSCM, including fluctuations in customer demand, insufficient supplier capacity, and dependence on exclusive suppliers, and has made specific adjustments and optimizations to the FCE model based on this comprehensive review.

In selecting risk factors and configuring model parameters, this study adheres to two fundamental principles. Firstly, it ensures that the chosen risk factors comprehensively cover the critical aspects of GSCM. Secondly, it guarantees that the parameter settings are both scientifically grounded and relevant to practical applications. To achieve these objectives, the study draws on advanced research findings from the aforementioned scholars and integrates insights from enterprise operational practices and expert consultations. This approach ensures that the selection of risk factors and the configuration of model parameters are well-supported by both theoretical and empirical evidence. For instance, indicators such as supplier diversity and supply chain transparency are incorporated into the model, informed by the latest research in GSCM and empirical evidence from industry practices.

**Fig. 2:**
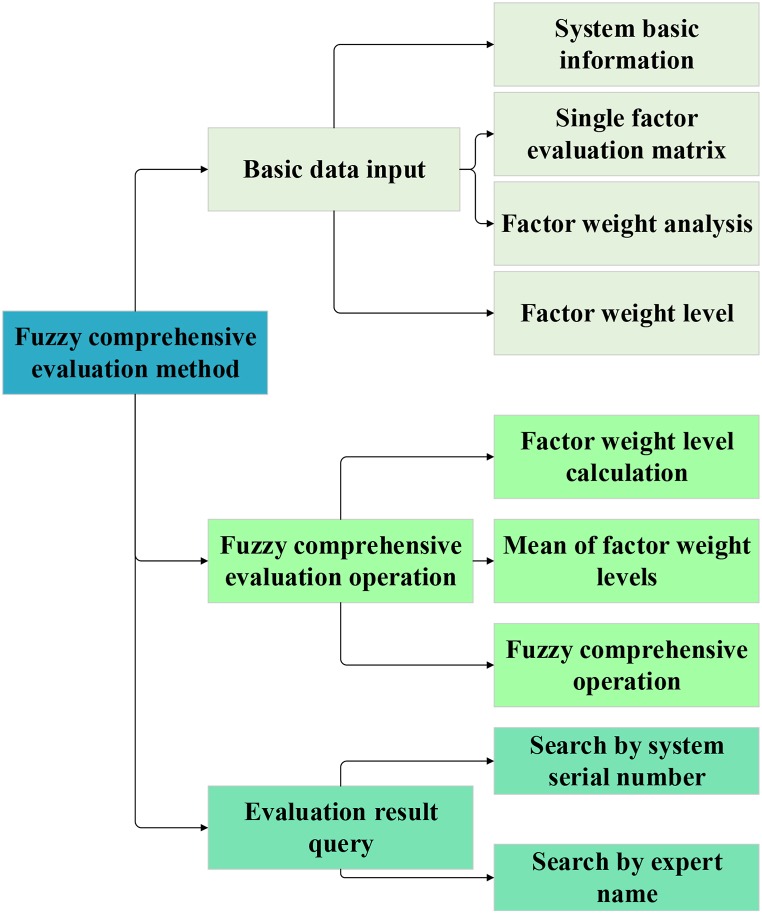
The architecture of the FCE model.

The FCE model used in this study is designed to assess the risk levels associated with intelligent management in GSCM. The model construction process begins by identifying evaluation indicators and their corresponding levels, and then dividing the value ranges of each indicator into several fuzzy subsets. Risk levels are categorized into “low risk,” “medium risk,” “high risk,” and so forth, with each subset described by a membership function representing its degree of membership. A single-factor evaluation is conducted for each indicator, determining the membership degree at each level based on actual conditions. This evaluation can be supported by expert judgment, historical data, or fuzzy statistical methods. Additionally, the weights of each evaluation indicator are established to reflect their relative importance in the overall assessment. Weight calculation may be performed using methods such as the AHP or the FCE method, considering the relationships and importance among various indicators. Using the membership degrees and weights of each evaluation indicator, the comprehensive membership degree for each subset under various indicators is calculated, thus determining the overall membership degree of each subset in the final evaluation. Based on the comprehensive evaluation results, the final risk level is determined by mapping the comprehensive membership degrees to the corresponding risk levels according to predefined criteria or standards. This mapping process establishes the risk level for corporate GSCM. The theoretical foundation for constructing the FCE model is rooted in fuzzy mathematics theory. This theory introduces factors of uncertainty and fuzziness into the evaluation process, aiming to produce results that more accurately reflect real-world conditions. Mathematically, the FCE model involves fuzzy set theory, fuzzy logical operations, fuzzy similarity measurement, and related areas. By performing mathematical operations on the membership degrees and weights of each evaluation indicator, the model produces a final risk level assessment for corporate GSCM. The FCE method, grounded in fuzzy mathematics, quantifies factors with unclear boundaries or difficult definitions. It is primarily used to address problems characterized by significant fuzziness and uncertainty in real-world environments. The model's architecture is illustrated in Fig. 2 [[49], [50], [51]].

The construction of the FCE model is divided into five steps.

Step 1: Fuzzy sets are established based on the study object. The evaluated index is set with the corresponding factor set $A=\left\{A_{1},A_{2},\cdots ,A_{n}\right\},i=1,2,\cdots ,n$. *n* is the number of first-level evaluation indicators $A_{i}$. $A_{i}=\left\{a_{i1},a_{i2},\cdots ,a_{im}\right\},j=1,2,\cdots ,m$, *m* is the number of secondary indicators $a_{ij}$. The remark set $B=\left\{B_{1},B_{2},\cdots ,B_{c}\right\}$ is set, where $B_{k}$ is the result of the evaluation, k=1,2,⋯,c. *c* is the number of evaluation levels.
Step 1: The single factors are evaluated, and the judgment matrix *S* is obtained. Each relevant factor is used to determine the membership degree of each evaluation level, that is, a fuzzy mapping *f: A→B* from the set *A* to *B* to find the evaluation matrix $S_{i}$ of $A_{i}$, as shown in Eq. (1):

(1) $$S_{i}={\left(s_{ijk}\right)}_{m*c}=\left[\begin{array}{ccc} s_{i11} & \cdots & s_{i1c}\\ \vdots & \ddots & \vdots \\ s_{ij1} & \cdots & s_{ijc} \end{array}\right],i=1,2,\cdots ,n$$

Eq. (1) represents the membership degree of the index $a_{ij}$ to *k*-level remark $B_{k}$. In index $a_{ij}$, if there are $h_{c}$ $B_{c}$ comments, $s_{ijk}$ can be confirmed using Eq. (2).

(2) $$s_{ijk}=h_{c}/\sum_{h-1}^{c}h_{k}$$

Step 3: The index weights at all levels are calculated. Rough set theory constructs an information system by establishing a sample and index evaluation system. Then, each index's importance obtains relevant weights [52,53]. Firstly, the information system is constructed, the evaluation index system is used as the attribute set in the information system, and the sample is selected as the domain. Secondly, the importance equation is employed to calculate each index's importance. Finally, Eq. (3) is utilized to calculate the weight value of each index of design evaluation [54].

(3) $$Q_{i}=Sig\left(i\right)/\sum_{i=1}^{n}Sig\left(i\right)$$

$Q=\left(Q_{1},Q_{2},\cdots ,Q_{n}\right)$ represents the weight vector of the indicator.

Step 4: It is a comprehensive evaluation, which is carried out in two steps. In step 1, the first-level indicator membership vector is calculated. Based on the single factor evaluation and index weight confirmation, the fuzzy algorithm is done on the evaluation matrix of the second-level index $a_{ij}$, and the membership vector of the related first-level evaluation index $A_{i}$ to the comment set $B_{j}$ is obtained:

(4) $$W_{i}=Q_{i}*S_{i}=\left(w_{i1},w_{i2},\cdots ,w_{ic}\right)$$

In Eq. (4), $Q_{i}$ is the weight matrix of secondary indicators, $Q_{i}=\left(q_{i1},q_{i2},\cdots ,q_{im}\right)$. $w_{ic}$ represents the membership degree of the first-level evaluation index to the comment $B_{k}$. In step 2, the membership vectors of the factor set are computed. The first-level indicators are collated to obtain the comprehensive matrix, as illustrated in Eq. (5):

(5) $$S=\left(\begin{array}{c} \begin{array}{c} W_{1}\\ W_{2} \end{array}\\ \begin{array}{c} \cdots \\ W_{n} \end{array} \end{array}\right)=\left(\begin{array}{cc} \begin{array}{cc} w_{11} & w_{12}\\ w_{21} & w_{22} \end{array} & \begin{array}{cc} \cdots & w_{1c}\\ \cdots & w_{2c} \end{array}\\ \begin{array}{cc} \cdots & \cdots \\ w_{n1} & w_{n2} \end{array} & \begin{array}{cc} \cdots & \cdots \\ \cdots & w_{nc} \end{array} \end{array}\right)$$

The calculation of the membership vector of a factor set A to B reads:

(6) $$W=R*S=\left(r_{1},r_{2},\cdots ,r_{n}\right)*{\left[W_{1},W_{2},\cdots ,W_{n}\right]}^{T}=\left(w_{1},w_{2},\cdots ,w_{c}\right)$$

In Eq. (6), $R=\left(r_{1},r_{2},\cdots ,r_{n}\right)$ is the weight matrix of the first-level index. $w_{c}$ represents the membership degree of the first-level evaluation index to the remark $B_{k}$.

Step 5: The results are evaluated. For first-level indicator $A_{i}$ pair of comment set $B_{j}$’s membership vector $v=\left(v_{i1},v_{i2},\cdots ,v_{ic}\right)$, and the membership vector of a factor set *A* to comment set *B* is assessed according to the principle of the maximum membership vector. The evaluation result is obtained, as follows in Eq. (7).

(7) $$v^{*}=\max\;\left\{v_{1},v_{2},\cdots ,v_{c}\right\}$$

Finally, the positional value of the comment set corresponding to the maximum value in each vector of b∗ is utilized.

The steps of FCE algorithm are shown in Table 2:

**Table 2:** Steps of FCE algorithm.

Step	Description
*1*	Initialization: Set the evaluation set $U=\left\{u_{1},u_{2},\cdots ,u_{n}\right\}$, the evaluation factor set $V=\left\{v_{1},v_{2},\cdots ,v_{m}\right\}$, and the evaluation level set $G=\left\{g_{1},g_{2},\cdots ,g_{l}\right\}$
*2*	Construct a single factor evaluation matrix: for each factor $v_{ij}$, construct its membership matrix $r_{ij}$ at each evaluation level
*3*	Determine the weight of each factor: use methods (such as hierarchical analysis method, expert scoring, etc.) to determine the weight $\omega_{j}$ of each factor in the factor set V
*4*	Perform a single factor evaluation on each factor, determine its membership and multiply it by the corresponding weight
*5*	Synthesize the evaluation of each factor to obtain the comprehensive membership $\psi_{il}$ of each evaluation object for each evaluation level
*6*	Normalize the comprehensive membership to obtain the normalized membership $\psi_{il}^{\prime }$
*7*	Determine the final evaluation level $g_{i}$ of the evaluation object based on the normalized membership
*8*	Output the evaluation result: take the final evaluation level $g_{i}$ of the evaluation object as the output

In the field of GSCM, adapting the fuzzy evaluation model is crucial, requiring a robust theoretical foundation and empirical evidence for its selection and configuration. Initially, the selection of risk factors is guided by authoritative research within the field. For instance, Yildiz Çankaya et al. (2019) identified key risk factors in GSCM, including changes in environmental regulations, the environmental performance of suppliers, and the environmental impact of product lifecycles [55]. To ensure the scientific validity and accuracy of these factors, this study also references the comprehensive review of GSC performance indicators conducted by Rosyidah et al. (2022) [56], which provides a concrete framework and standards for their selection.

In configuring the parameters of the fuzzy evaluation model, this study relies on classical literature in fuzzy set theory and fuzzy logic control. For example, the foundational theoretical support for the fuzzy evaluation model is derived from the fuzzy set theory outlined by Jasiulewicz-Kaczmarek et al. (2021) [57], while methodological guidance for parameter configuration is informed by Işık's (2023) fuzzy logic system design theory [58]. Specifically, when determining the shape and parameters of the membership function, this study draws on the application research of the FCE model in supply chain risk assessment by Wang et al. (2021) [59], which provides design ideas and methods for parameter adjustment.

In elaborating on the theoretical support for the selected risk factors and parameters, this study highlights their empirical validation in existing literature. For example, Kalyar et al. (2020) demonstrated that changes in environmental regulations significantly impact SCM, with suppliers' environmental performance being a crucial predictor of GSC performance [60]. Yu et al. (2022) further emphasized the critical role of product lifecycle management in GSC [61]. These studies offer a solid theoretical foundation and empirical support for the chosen risk factors. Furthermore, regarding the configuration of model parameters, this study references the research on fuzzy risk assessment methods by Ge et al. (2021) [62], which validates the effectiveness of the fuzzy evaluation model in managing uncertainty and provides practical evidence for parameter selection.

### 3.3 Analysis of SCM risk factors

The complexity inherent in the GSC system highlights the inevitability of supply chain risks from both practical and theoretical perspectives. The vulnerability of the supply chain, coupled with the uncertainties of the external business environment, can render an enterprise's supply chain operations susceptible to risks, potentially leading to inefficiencies or failures [63,64]. SCRM primarily involves identifying and evaluating various internal and external risk factors within the supply chain, and implementing management measures tailored to these specific risks. This management process integrates into the operational framework of the enterprise's supply chain and spans multiple domains [65]. SCRM is fundamentally achieved through the processes of identification, analysis, assessment, resolution, control, and prevention, all of which are intricately interconnected [66].

**Fig. 3:**
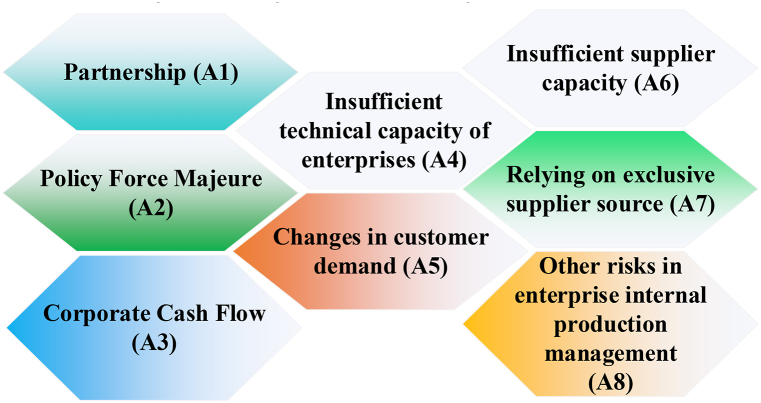
Risk factors of SCM of Company Y.

To illustrate this, Company Y, a player in the rail transit equipment manufacturing industry, serves as a case study to analyze risk factors in SCM. This analysis examines the development status and industry characteristics of the rail transit equipment manufacturing sector. Additionally, the current situation of Company Y and the attributes of its GSC are investigated, categorizing its supply chain risk factors into eight distinct categories, as illustrated in Fig. 3.

In Fig. 3, the risk factors are derived and summarized based on relevant research literature and expert interviews concerning intelligent management risks within GSC. Moktadir et al. (2021) introduced the concept of intelligent SCM, identifying risk factors across five dimensions: social, environmental, economic, technological, and institutional [67]. In contrast, Chu et al. (2020) categorized intelligent management risks in GSC into four primary areas: supply risks, process and control risks, environmental and sustainability risks, and demand risks [68]. Building on a synthesis of previous scholarly research and consultations with domain experts, this study defines eight key aspects to characterize the risks associated with intelligent management in GSC: partnership (A1), policy force majeure (A2), corporate cash flow (A3), enterprise technical capacity deficiency (A4), customer demand changes (A5), supplier capacity deficiencies (A6), dependence on exclusive supplier sources (A7), and internal production management risks (A8) [69,70]. These factors span a broad spectrum, including social, economic, institutional, technological, supply, process control, and environmental considerations. Further refinement of these factors is based on the literature. For example, A1 encompasses issues such as insufficient information sharing between supply chain partners (A11), the need to optimize supply chain structure (A12), weak risk awareness among suppliers (A13), disruptions caused by personnel flow in the supply chain (A14), and inadequate risk control for new products transitioning from pilot to mass production (A15). A2 includes risks such as production halts due to political force majeure (A21), transportation restrictions due to frequent environmental issues (A22), accidents during production (A23), and significant changes in national industrial operation regulations (A24). A3 encompasses several issues related to financial and logistical aspects, including extended transportation times (A31), rising costs of purchased products (A32), cash flow problems stemming from excessive inventory (A33), and delays in goods delivery due to payment issues influenced by the geographical distribution of suppliers (A34). A4 addresses challenges related to technical and design aspects, such as frequent changes in product design schemes (A41), a low degree of product standardization (A42), and a high dependency of enterprise products on strategic partners (A43). A5 highlights issues related to customer demand, including insufficient production cycles due to fluctuating customer demands (A51) and uncertainty in product technical requirements resulting from customer needs (A52). A6 pertains to capacity-related issues, specifically the insufficient capacity of core suppliers (A61). A7 involves issues related to supplier relationships, such as having too few alternative suppliers or an excessive number of exclusive suppliers (A71). A8 focuses on internal production management risks, including failures of manufacturing equipment or damage to inspection equipment (A81), unreasonable safety inventory levels (A82), low efficiency in information communication due to misaligned supply chain objectives (A83), delays in first inspection procedures for purchasing samples and small batch production (A84), and a lack of early warning mechanisms for production planning (A85), among other concerns.

### 3.4 Legal regulations for intelligent management of GSC

In China, various legal regulations govern the intelligent management of GSC, providing directives, frameworks, and guidelines for enterprises to effectively integrate intelligent management practices into their production processes. The principal legal regulations are summarized in Table 3.

**Table 3:** Legal regulations for intelligent management of GSC.

Legal Regulation	Main Content
*Environmental Protection Law*	It outlines environmental protection requirements that enterprises should adhere to in their production operations, including regulations on waste emissions, pollutant treatment, and environmental impact assessment.
*Resource and Environment Planning Law*	It specifies principles and requirements for resource and environmental planning, encouraging the rational development and utilization of resources while protecting the ecological environment.
*Circular Economy Promotion Law*	It promotes the development of a circular economy and encourages enterprises to engage in resource conservation, reuse, and recycling, aiming to reduce resource waste.
*Clean Production Promotion Law*	It encourages enterprises to adopt clean production technologies to reduce environmental pollution and enhance production efficiency.
*Environmental Information Disclosure Law*	It specifies principles for disclosing environmental information, requiring enterprises to disclose their environmental data, emission status, and other related information.
*Green Product Certification*	Green product certification system that allows enterprises to apply for certification to demonstrate the environmental advantages of their products.
*Energy Conservation and Emission Reduction Policies*	Policies aim to promote energy conservation and emission reduction, encouraging enterprises to decrease energy consumption and greenhouse gas emissions.
*Sustainable Development Strategies*	Sustainable development strategies and planning encourage enterprises to consider social, environmental, and economic benefits in their production operations.
*Corporate Social Responsibility Reporting System*	It promotes the submission of corporate social responsibility reports, including information about environmental protection and sustainable development efforts.

Table 3 illustrates the critical role of legal regulations in the intelligent management of GSC for enterprises. These regulations provide a structured framework, guidance, and constraints that compel enterprises to adopt intelligent management practices that promote sustainability within their GSC. They outline the environmental responsibilities and obligations of enterprises, incentivizing measures to reduce pollutant emissions, optimize waste disposal, and ensure environmental integrity in supply chain operations. Additionally, the regulations mandate the rational use of resources in production processes and the optimization of resource allocation through intelligent management, thereby minimizing waste and environmental impact. Furthermore, they promote transparency by encouraging enterprises to disclose environmental data; intelligent management facilitates accurate data collection and analysis, enhancing transparency. The regulations also advocate for reductions in energy consumption and carbon emissions, with intelligent management techniques aiding enterprises in optimizing supply chain operations, transportation logistics, and other areas to minimize their carbon footprint. Additionally, these regulations support enterprises in the prompt identification and evaluation of various supply chain risks, providing a structured framework for effective risk mitigation.

## 4 Datasets collection, experimental environment, and parameters setting

In conducting the questionnaire survey for this study, the research follows a stringent timeline and execution standards. The questionnaire design phase takes approximately two weeks to ensure comprehensive coverage of all relevant aspects of the research, with scientifically and rationally formulated questions. Before the official release, a one-week pilot test is conducted to assess the validity and clarity of the questionnaire, leading to necessary adjustments. The formal survey lasts four weeks, during which questionnaires are distributed to and collected from the target sample group. After data collection, about three weeks are spent on data organization, entry, and analysis. To ensure the accuracy and reliability of the results, a two-week validation period follows, during which consultations and discussions with industry experts are conducted.

The questionnaire is developed based on the analysis of SCM risk factors for Company Y and consists of three sections: (1) basic background information of the respondents, (2) perceptions of the fundamental situation of enterprise supply chain risk management (SCRM), and (3) assessment scores of Company Y's supply chain risk factors. The severity of each risk factor is categorized into five levels, with higher numerical values indicating greater risk levels. To enhance the credibility and precision of the research outcomes, this study combines the fuzzy decision-making method with actual data obtained from the questionnaire, using Company Y as a case study to validate the proposed model. The collected data are analyzed and processed using SPSS 2016, and the model's validity is further verified through expert consultation and email communication. The respondents are primarily senior management personnel from Company Y's supply chain departments. Out of 200 distributed questionnaires, 172 responses are received. After excluding incomplete or uniformly answered questionnaires, 164 valid responses are retained for analysis. The data collection status is detailed in Table 4.

**Table 4:** Dataset collection.

-	Number of people	Data characteristics description (number of people)			
Y Company Intelligent Supply Chain Management Department	247	Department head (1)	Senior manager (18)	Mid-level manager (86)	Junior manager (143)
Questionnaire Survey	200	Department head (1)	Senior manager (18)	Mid-level manager (57)	Junior manager (88)

Table 4 presents the structure of Company Y's intelligent SCM department, which comprises 247 individuals, including one department head, 18 senior managers, 86 mid-level managers, and 143 junior managers. In this survey, 200 questionnaires are distributed, resulting in an 81 % response rate. Participation is comprehensive, including all senior managers and department heads, along with 57 mid-level managers and 88 junior managers, thus ensuring the inclusivity and reliability of the dataset.

To validate the model's effectiveness, the verification process is rigorously described. Initially, the model's input-output relationship is examined through statistical analysis, with its predictive capability and accuracy assessed via correlation and regression analyses. Subsequently, reliability tests are conducted, including internal consistency checks and retest reliability analyses, to confirm the model's stability and repeatability. For instance, the internal consistency of each risk factor in the model is evaluated using Cronbach's α coefficient, which is calculated as shown in Equation (8):

(8) $$\alpha =\frac{N∙\bar{Cov}}{V_{t}+\left(N-1\right)∙\bar{Cov}}$$

N represents the total number of items in the questionnaire, $\bar{Cov}$ is the average covariance between the scores of all items, and $V_{t}$ is the total variance of all item scores. The coefficient of determination (R^2^ value) for regression analysis is calculated as shown in Equation (9):

(9) $$R^{2}=1-\frac{{SS}_{res}}{{SS}_{tot}}$$

${SS}_{res}$ denotes the sum of squared residuals, which is the sum of the squares of the differences between actual observed values and model-predicted values. ${SS}_{tot}$ represents the total sum of squares, which is the sum of the squares of the differences between actual observed values and their mean. Sensitivity analysis typically involves varying the input parameters of the model and observing the resulting changes in output. It is used to assess the degree of sensitivity of the model to changes in parameters.

The results show that Cronbach's α coefficient is above 0.7, indicating that the model has high reliability. In addition, the model's sensitivity analysis is also carried out to evaluate the influence of different parameter settings on the model output, and by comparing the model output results under different parameters, the model is robust and reliable in various situations. Part of the statistical results of the model validation process are listed in Table 5:

**Table 5:** Part of the statistical results of the model validation process.

Indicator	Value	Explanation
Cronbach's α coefficient	0.85	It indicates high internal consistency of the model.
Regression analysis of R^2^ value	0.78	It indicates that the model has strong predictive ability.
Sensitivity analysis results	Small range of variation	It indicates that the model has good robustness to parameter changes.

This study initially defines the indicator system for supply chain risk assessment based on data collected from the questionnaire survey. The weight $\omega_{i}$ of each risk factor is determined through expert scoring and the AHP. The scores $s_{i}$ for each risk factor are quantified based on the survey results and expert experience. The experiment calculates the comprehensive score for each risk factor using the following equation, and further assess the overall risk level of the supply chain. The comprehensive score for each risk factor is given by Equation (10):

(10) $$M_{i}=\sum_{j=1}^{m}\left(\omega_{ij}\times s_{ij}\right)$$

m represents the number of evaluation levels, $\omega_{ij}$ is the weight of the i-th risk factor at the j-th evaluation level, and $s_{ij}$ is the score at the corresponding level. The study further utilizes these comprehensive scores to convert qualitative assessments into quantitative analysis using fuzzy mathematics methods, thereby providing a more accurate evaluation of the supply chain's risk level.

## 5 Experimental results

### 5.1 Questionnaire survey results

**Fig. 4:**
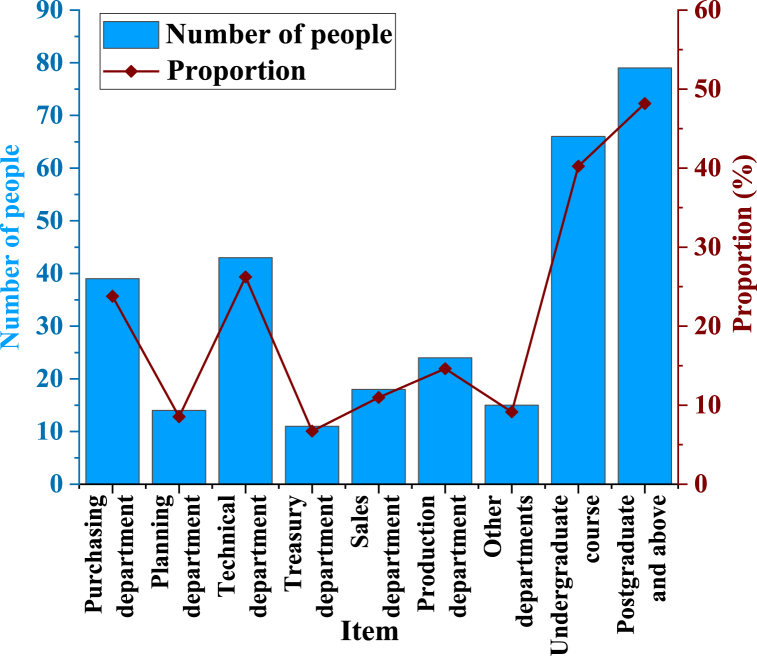
Basic information of the respondents.

The questionnaire is used for basic personnel statistics, as indicated in Fig. 4.

Fig. 4 illustrates that the majority of respondents are from the technical department, comprising 43 individuals, which represents 26.22 % of the sample. The procurement department follows closely with 39 respondents, accounting for 23.78 % of the sample. In contrast, the warehouse management department has the smallest representation, with only 11 individuals, or 6.71 % of the total sample. Concerning educational qualifications, 66 respondents hold bachelor's degrees, reflecting 40.24 % of the cohort. Additionally, 79 respondents possess graduate degrees or higher, constituting 48.17 % of the sample. Overall, 88.41 % of respondents hold university degrees or higher.

**Fig. 5:**
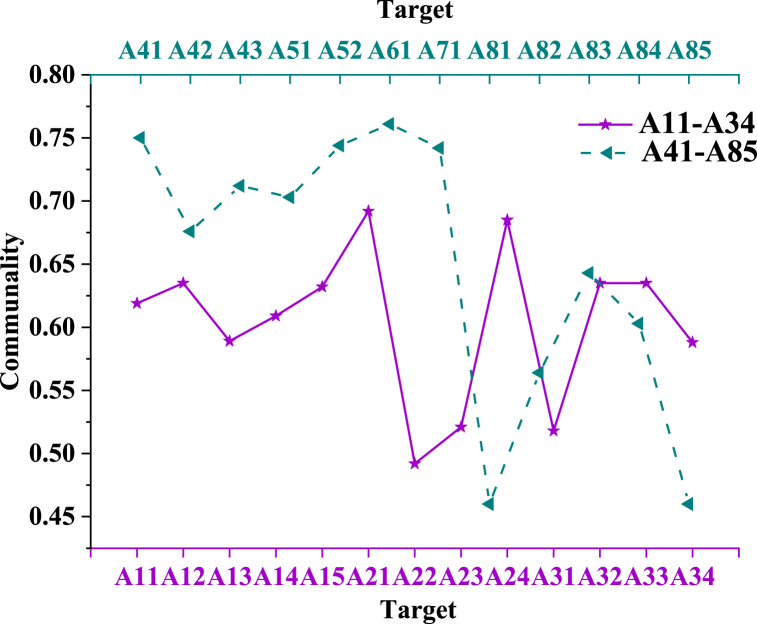
Common results of various factors.

The criterion for factor deletion is based on the concept of commonality. Commonality coefficients measure the degree of correlation between each observed variable and the principal components, indicating the proportion of total variance explained by each variable. Observed variables are grouped into distinct principal components to reduce data dimensions. When applying commonality coefficients as a criterion for excluding risk assessment factors, a lower coefficient in principal component analysis suggests that the factor explains less variance in the principal component and has a weaker correlation with it. This implies that the factor may contribute minimally to the overall risk assessment. Chu et al. (2020) proposed using a threshold of 0.2 as an evaluation standard for effective factors in supply chain risk management (SCRM), which has been validated in subsequent research [71]. Consequently, factors with a commonality coefficient below 0.2 are considered to have insufficient explanatory power for assessing intelligent management risk in GSC and are therefore removed. Data statistics and Statistical Product and Service Solutions (SPSS) software are employed to analyze the commonality of 25 sub-factors, as shown in Fig. 5.

Fig. 5 shows that among the 25 sub-factors, A81, which refers to the failure of manufacturing equipment or damage to detection equipment, has the lowest commonality value of 0.46. In contrast, A61, related to the insufficient capacity of core suppliers, exhibits the highest commonality at 0.761. In principal component analysis, a lower commonality value indicates a weaker suitability of the variable, while a higher commonality value signifies a stronger correlation with other factors. Since all sub-factors have commonality values above 0.2, they are retained for further analysis. Following this, expert opinions are solicited via email to validate the selection of sub-factors. This validation confirms that the set of 25 sub-factor indicators accurately represents the risks associated with intelligent management in GSC.

Rosetta software is utilized to calculate the importance and weight of each indicator, with the weights presented in Table 6.

**Table 6:** Weight results of secondary indicators.

Target	Weight	Target	Weight
A11	0.2	A41	0
A12	0.4	A42	0
A13	0.2	A43	1
A14	0.2	A51	0.5
A15	0	A52	0.5
A21	0.4286	A61	1
A22	0.4286	A71	1
A23	0.1429	A81	0.5
A24	0	A82	0
A31	0.7273	A83	0
A32	0.1818	A84	0
A33	0.0909	A85	0.5
A34	0		

**Fig. 6:**
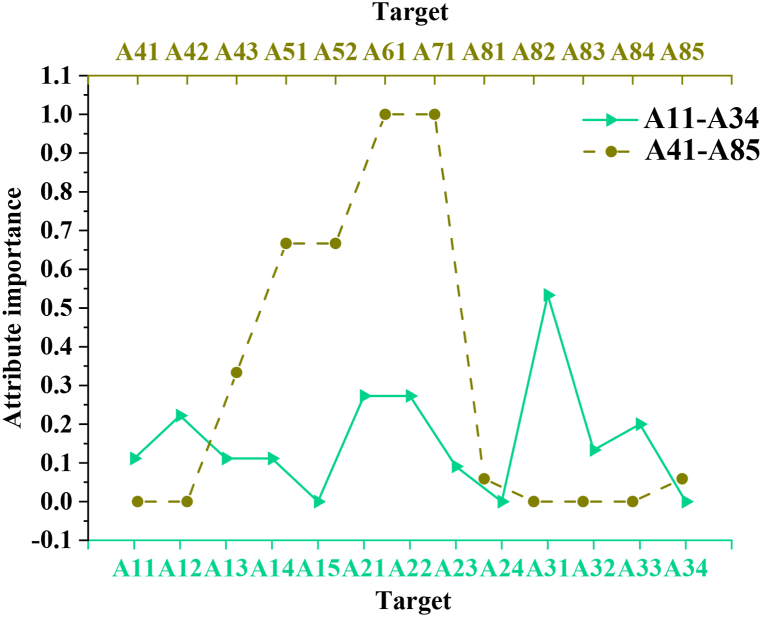
Results of attribute importance of secondary indicators.

Table 6 shows that not all indicators have weights exceeding 0. Specifically, under the A1 partnership indicator, the weight of A15, related to the transition of new products from pilot to batch production, is 0. Additionally, the weights of indicators A24, A34, A41, A42, A43, A82, and A84 are all 0. Notably, indicator A31, which pertains to prolonged transportation times due to the geographical dispersion of suppliers, has the highest weight at 0.7273. The attribute importance of each indicator is illustrated in Fig. 6.

**Fig. 7:**
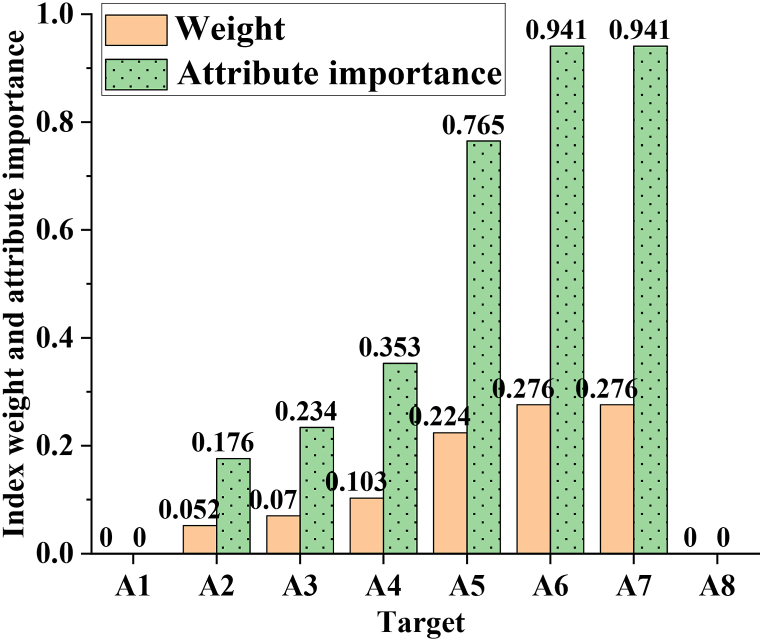
Weight and attribute importance of each first-level indicator.

Fig. 6 illustrates that indicators with a weight of 0 correspond to an importance score of 0. Despite indicator A31 having the highest weight, it is not the most critical, with an importance score of 0.5333. Instead, indicators A51 and A52 have the highest attribute importance scores, both at 0.6667. Fig. 7 presents a summary of the weights and attribute importance of each first-level indicator (A1-A8).

In Fig. 7, among the first-level indicators, A6 and A7 have the highest weight of 0.276, corresponding to the highest attribute importance score of 0.941. Conversely, indicators A1 and A8 bear a weight of 0 with negligible attribute importance. Notably, a positive correlation exists between weight and importance, indicating that importance also tends to increase as weight increases.

Finally, these factors are evaluated by FCE. Based on the actual operation of Company Y, the risk degree of its SCM is categorized into five levels: $B=\left\{b_{1},b_{2},b_{3},b_{4},b_{5}\right\}$ = {low risk, low risk, medium risk, high risk, high risk} = {1,2,3,4,5}, which is also the comment set in the FCE. Subsequently, the evaluation matrix S1-S8 of each first-level index is obtained, and the fuzzy calculation is executed for each second-level index. This process yields membership vectors for the factor set, and the evaluation outcome is determined according to the principle of maximum membership: *v*1∗ = 0.409; *v*2∗ = 0.334; *v*3∗ = 0.397; *v*4∗ = 0.360; *v*5∗ = 0.333; *v*6∗ = 0.317; *v*7∗ = 0.354; *v*8∗ = 0.308. Consequently, the evaluation outcomes illustrate that the risk of partnership, policy force majeure, cash flow, insufficient technical capacity, customer demand change, insufficient supplier capacity, relying on exclusive supplier sources, internal production management, and other risks are all classified under the evaluation b∗ = 3; b∗ = 3; b∗ = 3; b∗ = 3; b∗ = 4; b∗ = 4; b∗ = 4; b∗ = 3, indicative of medium risk for Company Y. Furthermore, the risks associated with customer demand fluctuations, insufficient supplier capacity, and reliance on exclusive supplier sources are classified under high risk.

Through a detailed analysis of the collected questionnaire data, this study can be found some high-risk factors for GSCM. These factors are mainly concentrated in the following aspects: enterprise internal management mechanism, coordination efficiency between upstream and downstream in the supply chain, and external policy environment. Firstly, internal management mechanisms within enterprises are pivotal for the effective implementation of GSCM. The survey data indicate that many enterprises lack robust green management mechanisms, particularly in fostering environmental awareness among employees and providing adequate green management training. This deficiency may stem from a lack of emphasis on GSCM within the enterprises, coupled with insufficient systematic training and incentive mechanisms, which impedes employees' ability to fully integrate environmental concepts into their practices. To address this issue, enterprises should consider implementing several measures, such as establishing GSCM incentive programs, conducting regular environmental training, and enhancing overall environmental awareness and skills. Additionally, formulating clear green management regulations can help ensure the effective execution of various environmental measures. Secondly, the efficiency of coordination between upstream and downstream segments of the supply chain is crucial for GSCM implementation. The survey results reveal that many enterprises experience issues related to information asymmetry and communication barriers in their interactions with suppliers and customers. These challenges not only impact the green operational efficiency of the entire supply chain but may also result in resource wastage and environmental pollution. Therefore, enterprises need to enhance communication and collaboration with suppliers and customers, establish information-sharing platforms, and strive for seamless integration across all segments of the supply chain. Furthermore, enterprises can enhance the effectiveness of GSCM by entering into GSC agreements and collaboratively establishing environmental goals and standards with upstream and downstream partners. This approach ensures synchronized advancements in green management practices across the entire supply chain. Additionally, the external policy environment plays a crucial role in the implementation of GSCM within enterprises. Analysis of the questionnaire data reveals that some enterprises either lack sufficient understanding of government environmental policies and regulations or face challenges in adapting to policy changes. These difficulties may arise from inadequate policy promotion or from the complexities involved in interpreting and implementing these policies. To address this issue, it is essential for the government to strengthen the promotion of environmental policies and provide targeted guidance and support to help enterprises better comprehend and adhere to these regulations. Concurrently, enterprises should proactively monitor policy developments and adjust their management strategies accordingly to remain compliant with policy requirements.

The analysis highlights that enterprises encounter multiple challenges in implementing GSCM. This study not only identifies these problems but also proposes corresponding mitigation strategies, ensuring the practical relevance of the research findings. By relating the results to broader trends and discussions within the GSCM field, it becomes apparent that these issues and solutions possess global significance and reference value. GSCM, as an internationally recognized environmental concept, has garnered widespread attention and application across various countries and regions. The challenges identified and the proposed solutions are consistent with international experiences and research, thereby contributing to the enhancement of green management practices and supporting the global advancement of GSCM. In summary, this study provides a detailed examination of high-risk factors and their underlying causes, along with specific mitigation strategies derived from an in-depth analysis of questionnaire data. The proposed analysis and recommendations are of significant practical value and offer substantial reference for enterprises and policymakers aiming to advance GSCM.

This study evaluates the risk levels associated with corporate GSCM using the FCE model and proposes a series of regulatory improvement recommendations. The analysis identifies several high-risk factors, including fluctuations in customer demand, inadequate supplier capacity, and dependency on exclusive suppliers. The discussion delves into the underlying causes of these high-risk factors, their specific impacts on business operations, and offers corresponding management recommendations.

Fluctuations in customer demand primarily result from uncertainties and unpredictabilities, such as changes in market trends, shifts in consumer preferences, and economic cycle variations. These fluctuations can lead to inventory excesses or shortages, thereby impacting supply chain efficiency and customer satisfaction. To address demand fluctuations, enterprises should implement flexible SCM strategies, including dynamic pricing, safety stock management, and advanced demand forecasting techniques. Inadequate supplier production capacity, influenced by factors such as technological limitations, financial constraints, or insufficient human resources, can hinder the ability to meet enterprise demands. This inadequacy can disrupt the supply chain, affecting production schedules, revenue, and company reputation. To mitigate these risks, enterprises should focus on enhancing supplier capacity through diversified supplier selection, establishing long-term partnerships, and providing technical support. Overreliance on exclusive suppliers, who may offer unique products or services, can increase supply chain risk. Issues such as production delays or quality problems with these exclusive suppliers can disrupt the supply chain. Enterprises should seek to diversify their supplier base and develop contingency plans to reduce the risks associated with reliance on exclusive suppliers. Therefore, enterprises should mitigate their reliance on single suppliers by diversifying their supplier base or developing alternative supply chains. Additionally, varying perspectives on risk factors across different departments can arise due to differing responsibilities and viewpoints. This variation highlights the necessity for cross-departmental communication and collaboration in formulating SCM strategies to ensure a cohesive understanding of risks and response measures. For instance, to reduce costs, enterprises might opt for suppliers with lower prices but also lower environmental standards, which conflicts with sustainability goals. Enterprises should strive to balance cost-effectiveness with sustainability by incorporating eco-friendly materials, enhancing production processes, and improving energy efficiency to achieve sustainable development.

The findings of this study emphasize the critical role of identifying and evaluating risk factors within GSCM. A comprehensive analysis of high-risk factors enables enterprises to develop more effective risk management strategies, thereby enhancing the resilience and sustainability of the supply chain. Concurrently, it is essential for enterprises to align risk management with sustainability objectives to ensure long-term business success and social value.

### 5.2 Cross-industry case analysis

Case 1: Electronics Manufacturing Industry
  Scenario: The supply chain in the electronics manufacturing industry frequently involves the extraction of rare metals, which can lead to environmental degradation and human rights concerns.
  Application: Contracts with suppliers mandate the provision of due diligence reports on conflict minerals to ensure that raw materials are sourced in a legal and ethical manner.
Case 2: Textile Industry
  Scenario: The textile industry encounters significant environmental issues, including wastewater management and chemical usage.
  Application: Contracts impose stringent restrictions on the use of chemicals and require suppliers to adopt environmentally friendly dyeing and processing techniques.
Case 3: Food and Beverage Industry
  Scenario: The food and beverage industry emphasizes sustainable agricultural practices and efforts to minimize food waste.
  Application: Contracts require the sourcing of raw materials from sustainable sources and encourage measures to reduce food waste across the entire supply chain.

Different industries face distinct challenges when implementing GSCM. For instance, heavy industries may be primarily concerned with energy consumption and emissions, while service industries might focus more on enhancing energy efficiency and reducing waste. Consequently, recommendations should be tailored to the specific needs of each industry. Heavy industries should prioritize reducing energy consumption and adopting clean technologies, whereas service industries should emphasize improving energy efficiency and minimizing operational waste. The agricultural sector should promote sustainable farming practices and the procurement of organic products. Potential obstacles and strategies for overcoming these challenges during the implementation process are outlined below.

Obstacle 1: Resource Constraints.
  Strategy: A phased implementation approach can be adopted, prioritizing investment in areas with the greatest impact. Enterprises can also seek government subsidies or funding support from partners to alleviate resource limitations.
Obstacle 2: Technological Challenges.
  Strategy: Collaborating with technology providers to acquire advanced environmental technologies and expertise is crucial. Additionally, investing in employee training to enhance technical capabilities will help address technological barriers.
Obstacle 3: Cultural Resistance.
  Strategy: Strengthening internal communication and raising employee awareness about the importance of GSCM are key to overcoming cultural resistance. Leadership demonstration can further facilitate the necessary cultural shift.

A comparison of enterprises of different sizes reveals that while larger companies may have more resources to implement GSCM, and SMEs can achieve significant results through flexible collaboration models and innovative partnerships. For instance, small enterprises can share resources by participating in industry associations, whereas large enterprises can leverage their scale to drive change across the entire supply chain. The characteristics of enterprises of varying sizes and types are summarized in Table 7.

**Table 7:** Enterprises of diverse sizes and types.

Enterprise type	Resource investment	Technology application	Cultural challenges	Collaboration mode	Implementation effect
Large enterprises	High	Advanced	Resistance to change	Supply chain cooperation	Remarkable
Medium-sized enterprises	Moderate	Moderate	Internal identification	Industry associations	Good
Small enterprises	Limited	Basic	Leadership	Partnership	Gradual improvement

Firstly, to enhance the practicality of the study, several specific implementation strategies and real-world examples are proposed: Strict Enforcement of Environmental Policies: Enterprises should establish dedicated environmental management departments responsible for formulating and overseeing the implementation of environmental policies. Additionally, collaboration with external environmental consulting firms for regular third-party audits is recommended. For instance, a major manufacturing company successfully reduces emissions and improves resource utilization efficiency by creating an environmental management department and partnering with a reputable environmental consulting firm. Establishment of GSC Information Disclosure Mechanisms: Enterprises need to develop or acquire SCM software to monitor and record environmental data from each segment of the supply chain in real-time. This data should be disclosed to the public regularly to enhance transparency. An example is an electronics manufacturer that implemented an advanced SCM system to comprehensively track its suppliers' environmental performance and issued quarterly environmental reports, thereby effectively enhancing its corporate social responsibility profile. Promotion of Clean Production Technologies: Enterprises should actively adopt and develop clean production technologies and provide relevant training to employees. They can also apply for government subsidies and preferential policies to offset the costs associated with technological upgrades. For example, a chemical company significantly reduces pollutant emissions by implementing advanced clean production processes and receives government financial support, which alleviating the economic burden of these technological enhancements. These strategies provide practical avenues for improving environmental management practices and can be adapted to various industry contexts to achieve effective GSCM.

Secondly, to enhance the general applicability of the research findings, a detailed examination of their relevance across various types of enterprises and industries is conducted: Manufacturing Industry: Given the complex supply chains and substantial resource consumption typical in manufacturing, the findings offer considerable reference value. By adopting intelligent management systems and adhering to legal regulations, manufacturing enterprises can effectively mitigate operational risks and enhance overall efficiency. Retail Industry: Although SCM in retail is generally simpler, it still encounters challenges such as inventory management and logistics distribution. The green comprehensive evaluation model proposed in this study is applicable to retail enterprises, aiding in the optimization of SCM processes and enhancing customer satisfaction. Technology Services Industry: Despite having less environmental pressure, technology service providers must still prioritize the sustainable development of their supply chains. The methods and recommendations presented in this study can assist technology service enterprises in selecting more environmentally friendly suppliers and improving their brand image. For enterprises of varying scales: Large Enterprises: With greater resources, large enterprises can more effectively implement intelligent management systems and regulatory measures. Their complex supply chain structures necessitate advanced risk assessment and management models to ensure efficiency and compliance. SMEs: Although SMEs may have limited resources, they can progressively adopt the recommended measures with the support of government policies and collaborations with third-party organizations. This approach can enhance their sustainability capabilities over time.

Finally, the broad applicability of the research results is further validated. Table 8 illustrates the applicability analysis results for enterprises of different sizes:

**Table 8:** The applicability analysis results of enterprises of different sizes.

Enterprise type	Applicability	Implementation difficulty	Success cases
Large manufacturing enterprises	High	Secondary	A large manufacturing enterprise
Small and medium-sized manufacturing enterprises	Secondary	High	A medium-sized chemical enterprise
Retail enterprises	Secondary	Low	An electronic product manufacturer
Technology service enterprise	Secondary	Low	A technology service enterprise

### 5.3 GSCM legal regulation suggestions

In the electronics manufacturing and textile industries, companies face significant challenges in ensuring ethical compliance and environmental sustainability within their supply chains. In the electronics manufacturing sector, conflict minerals—such as tantalum, tin, tungsten, and gold—present a major issue. These minerals often originate from conflict regions, and their extraction and trade may fund armed conflicts or involve serious environmental and human rights violations. Apple Inc. has proactively addressed this issue through several measures.

(1) Due Diligence Reports: Apple Inc. requires its suppliers to submit comprehensive due diligence reports, demonstrating that the sourcing of raw materials is legal and ethical. These reports include tracking records of mineral sources, audit results, and compliance certifications.
(2) Supply Chain Transparency: Apple Inc. has established a transparent supply chain tracing system to verify the origins of minerals. The company conducts regular third-party audits to ensure that suppliers adhere to relevant regulations and ethical standards.
(3) Conflict Minerals Reporting: Apple Inc. publishes an annual Supplier Responsibility Report, disclosing the usage of conflict minerals within its supply chain and detailing the measures taken to address this issue. These reports enhance supply chain transparency and showcase the company's commitment to ethical sourcing to the public and investors. Through these initiatives, Apple Inc. has not only reduced the use of conflict minerals but also promoted ethical and environmental compliance within its supply chain. This approach serves as a significant example for other companies, illustrating how to address ethical and environmental challenges in the supply chain through concrete legal and managerial policies. In the textile industry, the use of chemical dyes and the promotion of environmentally friendly materials are two primary concerns. Hennes & Mauritz AB (H&M), a leading global textile retailer, has implemented several measures to address these challenges and enhance the sustainability of its supply chain:
(1) Strict Supply Chain Management Policies: H&M has established stringent supply chain management policies requiring suppliers to reduce the use of chemical dyes and gradually eliminate hazardous chemicals. The company has implemented a “Chemicals Management System” to regulate the chemicals used by suppliers, ensuring compliance with global environmental standards.
(2) Use of Sustainable Materials: H&M actively promotes the use of environmentally friendly materials, such as organic cotton, recycled polyester, and other sustainable fabrics. The company has set “Sustainability Goals,” including converting all cotton used in its products to organic cotton to minimize environmental impact.
(3) Transparency and Public Reporting: H&M publishes an annual *Sustainability Report*, detailing its progress in environmental and social responsibility. These reports outline the company's achievements and future goals in reducing chemical dye usage and promoting sustainable materials.
(4) Collaboration and Certification: H&M collaborates with various environmental organizations to advance green supply chain certifications, such as Global Organic Textile Standard (GOTS) and Fair-Trade certification. Through these certifications, H&M ensures that its products meet high standards for environmental and social responsibility.
H&M's practices effectively reduce the use of chemical dyes in its supply chain and promote the adoption of sustainable materials. These efforts not only comply with environmental regulations but also enhance the company's brand image and market competitiveness.

Through the case studies of Apple Inc. and H&M, it is evident that companies can effectively address challenges in GSCM by establishing specific legal rules and management policies to tackle ethical and environmental issues. Apple Inc. ensures it does not engage in conflict minerals through due diligence and supply chain transparency, while H&M reduces environmental impacts through strict chemical management and the promotion of sustainable materials. These practices demonstrate how concrete measures and legal rules can facilitate sustainable supply chain development across different industries. The legal framework for GSCM must encompass a broad range of environmental and social standards while also considering specific legal challenges that companies may face in various jurisdictions. For instance, environmental regulations in different countries can have varying impacts on supply chain management. Companies need a thorough understanding and adaptability to these regulations to ensure global supply chain compliance. Moreover, transparency in supply chain information and traceability of environmental responsibilities are critical points in legal oversight. Companies should establish mechanisms to record and report the environmental impacts of their supply chains. This approach not only enhances the company's social responsibility image but also meets the increasing demands of consumers and regulatory agencies.

Compliance with relevant laws and regulations is essential for companies to avoid legal risks and economic losses. This study explores how companies can adhere to environmental regulations at different stages of the supply chain and illustrates the effectiveness of compliance management in reducing legal risks through case analyses. In the context of GSCM, legal regulation presents a complex and multifaceted challenge. To address the legal obstacles encountered by enterprises in GSCM, the following revised recommendations, informed by thorough research and case analysis, are proposed. These recommendations aim to enhance environmental compliance and sustainability, while offering practical guidance for policymakers in implementing GSCM.

1. Establishing Standardized GSC Contracts: Enterprises should develop standardized contract clauses for GSC that clearly delineate environmental responsibilities and compliance requirements for each segment of the supply chain. Contracts should mandate adherence to international environmental standards, such as ISO 14001, and require suppliers to provide proof of compliance. By standardizing these contract terms, enterprises can ensure that every component of the supply chain adheres to environmental regulations, thus mitigating legal risks. 2. Creating a Supply Chain Environmental Responsibility Traceability System: It is essential to implement a traceability system that tracks and evaluates the environmental impacts of each link within the supply chain. Such a system facilitates internal management and fulfills external regulatory requirements. It enables enterprises to promptly identify and address environmental issues, thereby enhancing the overall environmental performance of the supply chain. 3. Collaborating with Government Departments on Tax Incentive Policies: To encourage the adoption of environmentally friendly practices, enterprises should collaborate with government departments to advocate for tax incentives. Governments should provide tax benefits to enterprises implementing GSCM practices. By working together, specific tax incentive policies can be formulated to support GSCM initiatives. These policies can alleviate the financial burden on enterprises and promote broader participation in GSCM. 4. Promoting Interdisciplinary Research: Interdisciplinary research is crucial for developing a robust GSCM legal regulatory framework. Collaboration between experts in law, management, and environmental science is necessary to research and formulate applicable laws and policies. Through interdisciplinary efforts, a more comprehensive and scientifically grounded legal framework can be established, offering stronger guidance for enterprises.

By implementing these recommendations, enterprises can better achieve environmental compliance and sustainability, while policymakers can craft effective guidelines for advancing GSCM. Case studies serve as a crucial method for illustrating the practical application of GSCM legal regulations. By analyzing specific industry case studies, one can demonstrate how these regulations are effectively implemented in practice. For example, in the electronics manufacturing sector, the supply chain often involves the extraction of rare metals, which can lead to significant environmental and human rights concerns. To address this, contracts should mandate that suppliers provide due diligence reports on conflict minerals to ensure the legal and ethical sourcing of raw materials. Such case studies offer valuable references and examples for other enterprises. Moreover, industry-specific recommendations are essential. Different sectors face distinct challenges when implementing GSCM, which must be addressed with tailored approaches. For instance, heavy industries might emphasize issues related to energy consumption and emissions, while service industries may focus on improving energy efficiency and reducing waste. Therefore, recommendations should be customized to reflect the unique characteristics of each industry, providing relevant legal and regulatory advice to effectively address their specific needs. Furthermore, evaluating the effectiveness of GSCM implementation is a critical step in ensuring regulatory success. Enterprises should regularly assess the performance of their GSCM legal regulations and make necessary adjustments in response to evolving legal and market conditions. This can be accomplished through internal audits and third-party evaluations. Continuous effectiveness evaluations enable enterprises to refine their GSCM strategies, ensuring sustained environmental compliance and promoting long-term sustainable development.

The above recommendations offer practical guidance for enterprises in addressing legal challenges associated with GSCM. They also provide actionable insights for policymakers to implement GSCM effectively, thereby advancing the green transformation of various industries.

## 6 Limitation and discussion

This section, in conjunction with broader trends in the GSCM field, identifies the potential causes of high-risk factors and proposes possible mitigation strategies, thereby enhancing the study's practical application and academic contributions. Firstly, the study reveals that fluctuations in customer demand, inadequate supplier capacity, and dependence on exclusive suppliers are key risk factors in SCM. This finding is consistent with Liu et al. (2022), who identified similar risks in their analysis of the leather industry supply chain. Furthermore, the study highlights the significant impact of inefficient sewage treatment, shifts in consumer preferences, price and cost fluctuations, and fiscal changes on the successful implementation of sustainable SCM practices in emerging economies [72]. These factors underscore the vulnerability of enterprises in volatile market environments and changing policies, emphasizing the need for more adaptable and forward-thinking strategies when developing supply chain management approaches. Additionally, Karmaker et al. (2023), using Pareto analysis, fuzzy theory, and total interpretive structural modeling, investigated the interactions between supply chain risk factors. They identified major obstacles to sustainable development in SMEs, including lack of enthusiasm for sustainable practices among top management, insufficient technical capabilities, policy force majeure, and internal production management issues [73]. These findings suggest that achieving sustainable development requires not only technological innovation and enhanced production efficiency but also the active engagement and support of top management to ensure sustainability at both strategic and operational levels. Furthermore, Akter et al. (2022) explored the supply chain of emergency life-saving drugs, employing grey theory and decision experiment methods to identify and evaluate major threats such as financial and economic risks, shortages of critical raw materials, infectious disease outbreaks, and natural disasters [74]. These studies, alongside the present research, confirm that policy force majeure, inadequate supplier capacity, and dependence on exclusive suppliers remain among the most critical risks in the supply chain. This highlights the importance for enterprises to consider emergencies and uncontrollable factors when designing supply chain strategies and to develop more flexible and resilient supply chain systems.

The existing literature provides a solid foundation for GSCM risk assessment and legal regulation, though most studies have predominantly focused on qualitative analyses or traditional risk assessment methodologies. For example, Zou et al. (2021) developed a set of risk design principles and evaluation systems, utilizing grey relational analysis and backpropagation neural networks to assess GSC risks [75]. However, unlike the approach taken in this study, those scholars employed different analytical methods. In contrast, this study applies the FCE model, which not only addresses uncertainties and ambiguities in risk assessments but also offers a more systematic and comprehensive approach to analyzing supply chain risks, making risk evaluations more precise and effective. In applied research by Reshad et al. (2023) on sustainable SCRM in emerging economies such as Bangladesh, they identified and assessed barriers to sustainable SCM and prioritized key strategies. Their findings revealed that information-related obstacles and a lack of coordination were the most significant challenges, while strong top management commitment emerged as the most effective strategy [76]. However, many of these studies tend to overlook the interactions between risk factors and the overall complexity of the supply chain. The innovation in this study lies in the use of the FCE model, which not only focuses on individual enterprise-level risks but also considers the broader context of GSCM and legal regulation, providing a more holistic and integrated perspective. By employing the FCE model, this study introduces a novel quantitative risk assessment tool that enhances the existing body of knowledge by more accurately capturing the ambiguities and uncertainties inherent in supply chains. Furthermore, the study examines supply chain risks from multiple dimensions, including social, economic, environmental, and technological factors, providing new insights into the complex and multi-dimensional nature of these risks. From a practical standpoint, the FCE model enables enterprises to identify and assess potential supply chain risks, facilitating the adoption of appropriate risk mitigation strategies. In addition, the legal and regulatory recommendations offered in this study support enterprises in complying with environmental regulations, thereby mitigating legal risks and preventing economic losses associated with non-compliance. In summary, the adoption of the FCE model contributes both theoretical perspectives and practical tools, offering valuable guidance for corporate GSCM risk and environmental regulatory compliance. This enhances the resilience and sustainability of supply chains and plays a pivotal role in advancing the green transformation of the industry. The comprehensive analysis and insights provided herein not only enrich academic research but also offer practical value to corporate practice, thereby increasing both the academic and practical contributions of the study.

This study employs the FCE model to assess supply chain risks and proposes a series of targeted management recommendations and legal regulatory measures. Ji et al. (2022) utilized a system dynamics model and an improved FCE method to analyze the development trends of water resource carrying capacity [77]. Both methodologies share similarities in that they employ FCE to address uncertainties and ambiguities in evaluation problems. However, this study focuses on risk assessment in intelligent management within corporate GSCM, rather than analyzing the carrying capacity of a single resource. By using the FCE model, this research not only evaluates the risk levels within the supply chain but also integrates perspectives on environmental regulations and corporate sustainability, thereby offering more comprehensive management strategies and legal recommendations. This approach enhances the theoretical and practical applicability of the study. Additionally, Le (2023) explored the impact of GSCM on corporate performance and highlighted the role of big data capabilities [78]. While this study also examines corporate performance, it places greater emphasis on supply chain risk management. The empirical analysis identifies key risk factors within the supply chain and suggests corresponding risk mitigation measures. Moreover, this study underscores the importance of legal regulations in supply chain management, an aspect that Le's research did not extensively address. The integration of legal regulations with supply chain risk management provides enterprises with a more comprehensive risk management framework, facilitating sustainable supply chain development while complying with environmental regulations. This study not only enriches the theoretical knowledge base on supply chain risk assessment and management but also provides practical strategies that combine intelligent management with legal regulations. By comparing with existing literature, this research demonstrates its methodological and applicative innovation and forward-looking nature, offering new perspectives and research pathways for future studies.

This study provides a thorough analysis of risk assessment issues in corporate GSCM and proposes a series of targeted management strategies. However, it acknowledges several limitations that may affect the generalizability and depth of the findings. Firstly, while the FCE model excels at handling uncertainty and ambiguity, it may be less sensitive to dynamic changes in risk factors in specific contexts. Secondly, the data is primarily derived from a questionnaire survey of a single company, which may constrain the representativeness and applicability of the results. Additionally, while the study considers the impact of environmental regulations on supply chain management, it does not fully address the effects of regional cultural and policy differences on supply chain practices.

To address these limitations, future research could explore several directions: (1) employing cross-industry comparative studies to investigate the risk management needs of different sectors in GSCM; (2) examining companies from various regions to consider the impact of regional economic, political, and cultural differences on supply chain management; (3) delving into the application of intelligent management technologies in risk assessment and control, such as using machine learning to predict demand fluctuations; and (4) studying the construction and optimization of supply chain resilience and how risk management can enhance the supply chain's ability to withstand disruptions. These research directions could further enrich the theoretical and practical content of supply chain risk management, providing more effective risk management strategies for enterprises to achieve sustainable GSCM while adhering to environmental regulations.

The findings of this study have broad applicability across different industrial environments. In manufacturing, companies can use GSCM intelligent management risk assessment to identify and mitigate risks related to raw material supply, thereby reducing costs and improving production efficiency. For example, establishing long-term partnerships with suppliers can ensure stable raw material supply and mitigate risks associated with price fluctuations. In the retail sector, the study's results can help optimize inventory management strategies by more accurately forecasting market demand, reducing inventory backlog, and enhancing customer satisfaction. The GSCM risk assessment model proposed is applicable not only to manufacturing but also to other sectors, including services and information technology. In the services industry, the model can assist in evaluating and managing risks in the service supply chain, such as fluctuations in service quality and customer satisfaction. In the information technology sector, the model can be used to assess potential risks in the technology supply chain, such as insufficient supplier capabilities or rapid technological changes.

## 7 Conclusion

A company's sustainability strategy is crucial to its long-term success. Supply chain management plays a significant role in helping companies achieve their environmental, social, and economic goals. By adopting GSCM practices, companies can not only reduce their negative environmental impact but also enhance resource efficiency and achieve sustainable development. This study employs the FCE model to conduct a comprehensive assessment of intelligent management risks in GSCM and offers legal regulatory recommendations. The study identifies key high-risk factors in SCM, including fluctuations in customer demand, insufficient supplier capabilities, and reliance on exclusive suppliers. These factors negatively impact the stability and efficiency of a company's supply chain. Empirical analysis of Y Company's supply chain risks, based on questionnaire surveys and the FCE model, reveals that the company faces moderate to high-risk factors in its supply chain management. In response to these risks, the study proposes several improvement recommendations, such as strengthening the enforcement of environmental policies, establishing transparent supply chain information disclosure mechanisms, and promoting clean production technologies. These measures aim to enhance the resilience and sustainability of the supply chain. The theoretical and practical significance of this study lies in providing a new quantitative risk assessment tool that helps companies more accurately identify and evaluate potential risks in their supply chains and implement appropriate risk mitigation measures. Additionally, the study offers a multi-dimensional analysis of supply chain risks, providing new insights into the complexity of supply chain risk and contributing significantly to the green transformation of the entire industry.

The study not only offers new perspectives and tools for theory but also provides practical guidance for managing GSCM risks and ensuring environmental regulatory compliance. This contributes to strengthening the resilience and sustainability of supply chains and has significant implications for promoting the green transformation of the industry. To effectively mitigate risks in environmentally sustainable supply chains, this study recommends the following strategies:

Establish a comprehensive supply chain risk management framework to regularly assess and identify potential risk points.

Diversify supplier selection and establish long-term partnerships to reduce dependency on single suppliers.

Invest in clean production technologies and enhance energy efficiency to achieve both cost and environmental benefits.

Strengthen collaboration with government bodies and industry associations to leverage policy support and shared resources, enhancing the overall risk resilience of the supply chain.

By implementing these strategies, companies can improve their environmental performance, gain a competitive advantage in the market, and achieve sustainable development.

Based on these findings, the study presents a series of improvement recommendations aimed at enhancing GSCM. These recommendations include strengthening the enforcement of environmental policies, establishing transparent mechanisms for supply chain information disclosure, and promoting the adoption of clean production technologies. Implementing these suggestions can help enterprises achieve better compliance with environmental regulations and support their sustainable development efforts within GSCM. Furthermore, the study addresses the unique challenges encountered by different industries in implementing GSCM and provides industry-specific legal regulation recommendations. For instance, in the electronics manufacturing industry, it is recommended that contracts require suppliers to provide due diligence reports on conflict minerals to ensure the legality and ethical sourcing of raw materials. In the textile industry, contracts should impose restrictions on the use of harmful chemicals and mandate the adoption of environmentally friendly dyeing and processing technologies. In the food and beverage industry, contracts should focus on sustainable sourcing of raw materials and the reduction of food waste throughout the supply chain. These industry-specific case analyses offer practical operational references and provide policymakers with actionable guidelines for the effective implementation of GSCM. This study introduces new perspectives and tools for risk assessment and legal regulation within GSCM, contributing to the enhancement of supply chain resilience and sustainability. For policymakers, these findings offer robust support and guidance for advancing the green transformation of the entire industry, facilitating the integrated development of economic, social, and environmental objectives.

### 7.1 Theoretical and practical implications

From a theoretical standpoint, this study introduces the FCE model for a thorough assessment of risk levels in corporate GSCM. This theoretical advancement not only enhances the existing knowledge base within the SCM field but also provides new analytical tools for future research. The use of the FCE model represents a significant shift from previous reliance on qualitative analyses in supply chain risk assessments, offering a more accurate and dependable quantitative approach. Furthermore, the study examines the interplay between supply chain risk factors and environmental regulations, integrating legal regulation with risk management and thus providing novel theoretical insights into the multidimensional nature of supply chain risks.

Beyond its theoretical contributions, the study carries substantial practical implications. By analyzing the case of Company Y, the experiment validates the effectiveness of the FCE model in real-world SCM scenarios, demonstrating its capability to identify and evaluate supply chain risks. The findings highlight critical risk factors, including fluctuations in customer demand, inadequate supplier capacity, and reliance on exclusive suppliers. In response to these findings, the study offers several targeted management recommendations, such as diversifying supplier options, establishing long-term partnerships, and adopting clean production technologies. These recommendations aim to enhance the resilience and sustainability of supply chains in practical applications.

The relationship between theoretical significance and practical value is inherently complementary. Theoretical research offers methodological guidance and foundational principles that inform practical applications, while practical experiences validate these theories and stimulate their further development. Theoretical frameworks provide rich resources that can be applied in practice, and feedback from practical applications offers opportunities to test and refine theoretical models. This dynamic interaction not only enhances academic understanding of GSCM but also delivers actionable solutions for business managers navigating complex supply chain environments.

This study has made significant contributions on both theoretical and practical fronts. The rigorous theoretical research lays a robust foundation for practical application, while the successful implementation of these theories provides valuable case studies and data, enriching the theoretical framework. This reciprocal relationship ensures that the study remains both innovative and forward-looking in the academic realm and practical and instructive for real-world applications. As a result, the study offers comprehensive and effective support for corporate GSCM, bridging the gap between theory and practice.

### 7.2 Limitations and future work

However, there are certain limitations to the scope of GSC intelligent management addressed by this study. Given the multifaceted nature of GSCM, future research could focus on the following areas: Cross-Industry Comparative Studies: Investigate the different risk management needs across various industries to understand how GSCM practices can be adapted and applied in diverse contexts. Regional Variations: Explore how economic, political, and cultural differences impact GSCM risk management strategies in different regions. This will provide insights into how regional factors influence the effectiveness of GSCM practices. Application of Intelligent Management Technologies: Delve into the use of advanced technologies such as machine learning for risk assessment and control, particularly in predicting demand fluctuations and improving supply chain resilience. Supply Chain Resilience: Examine the construction and optimization of supply chain resilience, focusing on how risk management can enhance the supply chain's ability to withstand disruptions. These research directions aim to further enrich the theoretical and practical understanding of supply chain risk management, offering more effective strategies for businesses and supporting the sustainable development of GSCM while adhering to environmental regulations.

## Data availability statement

The datasets used and/or analyzed during the current study are available from the corresponding author Junfeng Wang on reasonable request via e-mail wangjunfeng@lnnu.edu.cn.

## Ethics statement

This article does not contain any studies with human participants or animals performed by any of the authors. All methods were performed in accordance with relevant guidelines and regulations.

## Declaration of competing interest

The authors declare that they have no known competing financial interests or personal relationships that could have appeared to influence the work reported in this paper.
