# Supplementary material for: Intelligent management and legal regulation of enterprise green supply chain by fuzzy comprehensive evaluation
Source: Heliyon. 2024 Oct 29;10(23):e39929. doi: 10.1016/j.heliyon.2024.e39929 (PMC11648754; doi:10.1016/j.heliyon.2024.e39929)
Supplement: Multimedia component 1 [file mmc1.docx]

**Questionnaire:**

Dear respondents,

Thank you for participating in this questionnaire survey. The research purpose is to gain a deeper understanding of the risk management intelligence level in Company Y's green supply chain management. Your opinions and suggestions are crucial to this study. Please carefully read each question and respond based on your actual perspectives.

**Section 1: Respondent Basic Information**

1. **Personal Background Information:**

- 1.1 Name:

- 1.2 Position:

- 1.3 Years of Work Experience:

- 1.4 Educational Background:

- 1.5 Department:

**Section 2: Awareness of Basic Situations in Corporate Supply Chain Risk Management**

2. **Basic Information about Corporate Supply Chain:**

- 2.1 Company Name:

- 2.2 Company Size (such as number of employees and annual revenue):

- 2.3 Primary Business of the Company:

- 2.4 Scope of the Supply Chain Coverage (such as suppliers, manufacturers, and distributors):

3. **Awareness of Corporate Supply Chain Risk Management:**

- 3.1 Are you familiar with the concept of corporate supply chain management?

A. Yes

B. No

- 3.2 Are you aware of the measures the company takes to manage supply chain risks?

A. Yes

B. No

- 3.3 Please briefly describe your understanding of corporate supply chain risk management:

**Section 3: Analysis and Rating of Supply Chain Risk Factors for Company Y**

4. **Rating of Supply Chain Risk Factors for Company Y:**

- 4.1 Please rate the following factors based on your understanding of Company Y, using a scale of five levels (1-5), where 1 indicates the lowest risk, and 5 indicates the highest risk.

- 4.1.1 Customer demand fluctuations: Rating ( )

- 4.1.2 Inadequate supplier capacity: Rating ( )

- 4.1.3 Reliance on exclusive supplier sources: Rating ( )

- 4.1.4 Partnerships: Rating ( )

- 4.1.5 Policies: Rating ( )

- 4.1.6 Corporate cash flow: Rating ( )

- 4.1.7 Insufficient technological capabilities: Rating ( )

- 4.1.8 Internal production management: Rating ( )

- 4.1.9 Other risks, please specify:

**Section 4: Fuzzy Comprehensive Evaluation and Empirical Research**

5. **Fuzzy Comprehensive Evaluation:**

- 5.1 Please assess the risk level of Company Y's intelligent supply chain management from your perspective (on a scale of 1-5, where 1 is the lowest risk and 5 is the highest risk). Rating ( )

**Section 5: Opinions and Suggestions**

6. **Opinions and Suggestions on Company Y's Supply Chain Risk Management:**

- 6.1 Based on your understanding, what issues do you think Company Y faces in supply chain risk management?

- 6.2 Drawing from your experience, provide recommendations to enhance and improve Company Y's supply chain risk management.

Thank you for your participation. Your opinions are crucial for the in-depth analysis and formation of conclusions in this study. Please answer the questions truthfully and in detail to ensure the accuracy of the survey information.
